# Response to “Vast (but avoidable) underestimation of global biodiversity”

**DOI:** 10.1371/journal.pbio.3001362

**Published:** 2021-08-13

**Authors:** Stilianos Louca, Florent Mazel, Michael Doebeli, Laura Wegener Parfrey

**Affiliations:** 1 Department of Biology, University of Oregon, Eugene, Oregon, United States of America; 2 Institute of Ecology and Evolution, University of Oregon, Eugene, Oregon, United States of America; 3 Biodiversity Research Centre, University of British Columbia, Vancouver, Canada; 4 Department of Botany, University of British Columbia, Vancouver, Canada; 5 Department of Zoology, University of British Columbia, Vancouver, Canada; 6 Department of Mathematics, University of British Columbia, Vancouver, Canada

## Abstract

This Formal Comment provides clarifications on the authors’ recent estimates of global bacterial diversity and the current status of the field, and responds to a Formal Comment from John Wiens regarding their prior work.

We welcome Wiens’ efforts to estimate global animal-associated bacterial richness and thank him for highlighting points of confusion and potential caveats in our previous work on the topic [1]. We find Wiens’ ideas worthy of consideration, as most of them represent a step in the right direction, and we encourage lively scientific discourse for the advancement of knowledge. Time will ultimately reveal which estimates, and underlying assumptions, came closest to the true bacterial richness; we are excited and confident that this will happen in the near future thanks to rapidly increasing sequencing capabilities. Here, we provide some clarifications on our work, its relation to Wiens’ estimates, and the current status of the field.

First, Wiens states that we excluded animal-associated bacterial species in our global estimates. However, thousands of animal-associated samples were included in our analysis, and this was clearly stated in our main text (second paragraph on page 3).

Second, Wiens’ commentary focuses on “S1 Text” of our paper [1], which was rather peripheral, and, hence, in the Supporting information. S1 Text [1] critically evaluated the rationale underlying previous estimates of global bacterial operational taxonomic unit (OTU) richness by Larsen and colleagues [2], but the results of S1 Text [1] did not in any way flow into the analyses presented in our main article. Indeed, our estimates of global bacterial (and archaeal) richness, discussed in our main article, are based on 7 alternative well-established estimation methods founded on concrete statistical models, each developed specifically for richness estimates from multiple survey data. We applied these methods to >34,000 samples from >490 studies including from, but not restricted to, animal microbiomes, to arrive at our global estimates, independently of the discussion in S1 Text [1].

Third, Wiens’ commentary can yield the impression that we proposed that there are only 40,100 animal-associated bacterial OTUs and that *Cephalotes* in particular only have 40 associated bacterial OTUs. However, these numbers, mentioned in our S1 Text [1], were not meant to be taken as proposed point estimates for animal-associated OTU richness, and we believe that this was clear from our text. Instead, these numbers were meant as examples to demonstrate how strongly the estimates of animal-associated bacterial richness by Larsen and colleagues [2] would decrease simply by (a) using better justified mathematical formulas, i.e., with the same input data as used by Larsen and colleagues [2] but founded on an actual statistical model; (b) accounting for even minor overlaps in the OTUs associated with different animal genera; and/or (c) using alternative animal diversity estimates published by others [3], rather than those proposed by Larsen and colleagues [2]. Specifically, regarding (b), Larsen and colleagues [2] (pages 233 and 259) performed pairwise host species comparisons within various insect genera (for example, within the *Cephalotes*) to estimate on average how many bacterial OTUs were unique to each host species, then multiplied that estimate with their estimated number of animal species to determine the global animal-associated bacterial richness. However, since their pairwise host species comparisons were restricted to congeneric species, their estimated number of unique OTUs per host species does not account for potential overlaps between different host genera. Indeed, even if an OTU is only found “in one” *Cephalotes* species, it might not be truly unique to that host species if it is also present in members of other host genera. To clarify, we did not claim that all animal genera can share bacterial OTUs, but instead considered the implications of some average microbiome overlap (some animal genera might share no bacteria, and other genera might share a lot). The average microbiome overlap of 0.1% (when clustering bacterial 16S sequences into OTUs at 97% similarity) between animal genera used in our illustrative example in S1 Text [1] is of course speculative, but it is not unreasonable (see our next point). A zero overlap (implicitly assumed by Larsen and colleagues [2]) is almost certainly wrong. One goal of our S1 Text [1] was to point out the dramatic effects of such overlaps on animal-associated bacterial richness estimates using “basic” mathematical arguments.

Fourth, Wiens’ commentary could yield the impression that existing data are able to tell us with sufficient certainty when a bacterial OTU is “unique” to a specific animal taxon. However, so far, the microbiomes of only a minuscule fraction of animal species have been surveyed. One can thus certainly not exclude the possibility that many bacterial OTUs currently thought to be “unique” to a certain animal taxon are eventually also found in other (potentially distantly related) animal taxa, for example, due to similar host diets and or environmental conditions [4–7]. As a case in point, many bacteria in herbivorous fish guts were found to be closely related to bacteria in mammals [8], and Song and colleagues [6] report that bat microbiomes closely resemble those of birds. The gut microbiome of caterpillars consists mostly of dietary and environmental bacteria and is not species specific [4]. Even in animal taxa with characteristic microbiota, there is a documented overlap across host species and genera. For example, there are a small number of bacteria consistently and specifically associated with bees, but these are found across bee genera at the level of the 99.5% similar 16S rRNA OTUs [5]. To further illustrate that an average microbiome overlap between animal taxa at least as large as the one considered in our S1 Text (0.1%) [1] is not unreasonable, we analyzed 16S rRNA sequences from the Earth Microbiome Project [6,9] and measured the overlap of microbiota originating from individuals of different animal taxa. We found that, on average, 2 individuals from different host classes (e.g., 1 mammalian and 1 avian sample) share 1.26% of their OTUs (16S clustered at 100% similarity), and 2 individuals from different host genera belonging to the same class (e.g., 2 mammalian samples) share 2.84% of their OTUs (methods in S1 Text of this response). A coarser OTU threshold (e.g., 97% similarity, considered in our original paper [1]) would further increase these average overlaps. While less is known about insect microbiomes, there is currently little reason to expect a drastically different picture there, and, as explained in our S1 Text [1], even a small average microbiome overlap of 0.1% between host genera would strongly limit total bacterial richness estimates. The fact that the accumulation curve of detected bacterial OTUs over sampled insect species does not yet strongly level off says little about where the accumulation curve would asymptotically converge; rigorous statistical methods, such as the ones used for our global estimates [1], would be needed to estimate this asymptote.

Lastly, we stress that while the present conversation (including previous estimates by Louca and colleagues [1], Larsen and colleagues [2], Locey and colleagues [10], Wiens’ commentary, and this response) focuses on 16S rRNA OTUs, it may well be that at finer phylogenetic resolutions, e.g., at bacterial strain level, host specificity and bacterial richness are substantially higher. In particular, future whole-genome sequencing surveys may well reveal the existence of far more genomic clusters and ecotypes than 16S-based OTUs.

## Supporting information

S1 Text Methods details on computing microbiome overlaps between animals. (PDF)

S1 Data Host taxonomies considered. (CSV)

S2 Data R code for computing microbiome overlaps. (ZIP)

S3 Data Computed Jaccard similarities. (CSV)
