# Supplementary material for: Response to “Vast (but avoidable) underestimation of global biodiversity”
Source: PLoS Biol. 2021 Aug 13;19(8):e3001362. doi: 10.1371/journal.pbio.3001362 (PMC8362970; doi:10.1371/journal.pbio.3001362)
Supplement: S1 Text — (PDF) [file pbio.3001362.s001.pdf]

Response to: Vast (but avoidable) underestimation of global biodiversity
# - Supplementary Material -

Stilianos Louca[1,2,*], Florent Mazel[3,4], Michael Doebeli[3,5,6] & Laura Wegener Parfrey[3,5,5]

[1]*Department of Biology, University of Oregon, Eugene, Oregon, USA*
[2]*Institute of Ecology and Evolution, University of Oregon, Eugene, Oregon, USA*
[3]*Biodiversity Research Centre, University of British Columbia, Vancouver, Canada*
[4]*Department of Botany, University of British Columbia, Vancouver, Canada*
[5]*Department of Zoology, University of British Columbia, Vancouver, Canada*
[6]*Department of Mathematics, University of British Columbia, Vancouver, Canada*
[*]Corresponding author

S.1 Computing microbiome overlaps between animals

Here we describe our analysis of prokaryotic OTU overlap between samples of animal gut microbiota, originating from individual animals of different genera. We used the 16S rRNA sequencing data set described by Song et al. [1]. This data set updates the Earth Microbiome Project data set (release #1) [2] with additional samples from vertebrate hosts for a total of 460 host species in 374 genera, mostly of birds and mammals. We provide a summary of the host diversity that was published in Song et al. [1] and that we used here in Supplemental File 1.

Details concerning the sampling, DNA extraction, PCR, sequencing and processing can be found in Song et al. [1] and follow the Earth Microbiome Project protocol [2]. Briefly, the V4 region of the 16S rRNA was amplified using the 515f/806r EMP primers and amplicons were sequenced on Illumina (MiSeq and HiSeq) platforms. Because the data set by Song et al. [1] originates from several studies, each respective data set was processed in the same way to produce an OTU table. Here, we did not used the raw fastq files but instead used the processed OTU tables produced by Song et al. [1] from the reads trimmed at 90pb. Those table are provided on the Qiita website (see details below). In the pipeline by Song et al. [1], reads were quality-filtered, checked against the Greengenes database [3] to remove artifactual sequences, and sequencing errors were removed using the deblur algorithm [4], yielding amplicon sequence variants (ASVs, i.e. 16S sequences clustered at 100% sequence similarity). The resulting ASV tables are publicly available and were downloaded from the Qiita website (`https://qiita.ucsd.edu/study/description/11166`) with Qiita biom table IDs being 93862,93855,93819,93914,93900,93846,93851 and 94483. Metadata are available for download as supplemental material of Song et al. [1] (DATA SET S1 therein) (`https://mbio.asm.org/content/mbio/11/1/e02901-19/DC1/embed/inline-supplementary-material-1.xlsx?download=true`).

Using the publicly available OTU tables and the publicly available metadata, we estimated the ASV overlap between samples of animal gut microbiota, originating from individual animals of different genera. The

R code that we used to produce these overlap estimates from the publicly available data is provided as Supplemental File 2 and is also available at `https://github.com/FloMazel/Overlap_Animal_Gut_Microbiota`. We proceeded as follow: first, we assigned taxonomy to the ASVs using the RDP classifier and the SILVA v132 database [5] and removed ASVs identified as Chloroplasts or Mitochondria. Second, we merged the ASV tables downloaded from the Qiita website and randomly selected one sample per host species. Third, we rarefied the data to 5000 reads per sample. Fourth, we computed overlap between samples using the presence/absence-based Jaccard metric [6].

We found that, on average, two individuals from different host genera belonging to different classes (e.g. one Mammalian sample and one Avian sample) share 1.26% of their ASVs and two individuals from different host genera belonging to the same class (e.g. two mammalian samples) shared 2.84% of their ASVs (Supplemental File 3).

References

[1] Song SJ, Sanders JG, Delsuc F, Metcalf J, Amato K, Taylor MW, et al. Comparative analyses of vertebrate gut microbiomes reveal convergence between birds and bats. MBio. 2020; 11(1). doi:https://doi.org/10.1128/mBio.02901-19.

[2] Thompson LR, Sanders JG, McDonald D, Amir A, Ladau J, Locey KJ, et al. A communal catalogue reveals Earth's multiscale microbial diversity. Nature. 2017; 551:457–463. doi:https://doi.org/10.1038/nature24621.

[3] DeSantis TZ, Hugenholtz P, Larsen N, Rojas M, Brodie EL, Keller K, et al. Greengenes, a chimera-checked 16S rRNA gene database and workbench compatible with ARB. Applied and Environmental Microbiology. 2006; 72(7):5069–5072. doi:https://doi.org/10.1128/AEM.03006-05.

[4] Amir A, McDonald D, Navas-Molina JA, Kopylova E, Morton JT, Xu ZZ, et al. Deblur rapidly resolves single-nucleotide community sequence patterns. MSystems. 2017; 2(2). doi:https://doi.org/10.1128/mSystems.00191-16.

[5] Glöckner FO, Yilmaz P, Quast C, Gerken J, Beccati A, Ciuprina A, et al. 25 years of serving the community with ribosomal RNA gene reference databases and tools. Journal of Biotechnology. 2017; 261:169–176. doi:https://doi.org/10.1016/j.jbiotec.2017.06.1198.

[6] Real R, Vargas JM. The probabilistic basis of Jaccard's index of similarity. Systematic Biology. 1996; 45(3):380–385. doi:https://doi.org/10.2307/2413572.
